# Sexually Antagonistic “Zygotic Drive” of the Sex Chromosomes

**DOI:** 10.1371/journal.pgen.1000313

**Published:** 2008-12-19

**Authors:** William R. Rice, Sergey Gavrilets, Urban Friberg

**Affiliations:** 1 Department of Ecology, Evolution, and Marine Biology, University of California Santa Barbara, Santa Barbara, California, United States of America; 2 Department of Ecology and Evolutionary Biology, University of Tennessee, Knoxville, Tennessee, United States of America; 3 Department of Mathematics, University of Tennessee, Knoxville, Tennessee, United States of America; Fred Hutchinson Cancer Research Center, United States of America

## Abstract

Genomic conflict is perplexing because it causes the fitness of a species to decline rather than improve. Many diverse forms of genomic conflict have been identified, but this extant tally may be incomplete. Here, we show that the unusual characteristics of the sex chromosomes can, in principle, lead to a previously unappreciated form of sexual genomic conflict. The phenomenon occurs because there is selection in the heterogametic sex for sex-linked mutations that harm the sex of offspring that does not carry them, whenever there is competition among siblings. This harmful phenotype can be expressed as an antagonistic green-beard effect that is mediated by epigenetic parental effects, parental investment, and/or interactions among siblings. We call this form of genomic conflict sexually antagonistic “zygotic drive”, because it is functionally equivalent to meiotic drive, except that it operates during the zygotic and postzygotic stages of the life cycle rather than the meiotic and gametic stages. A combination of mathematical modeling and a survey of empirical studies is used to show that sexually antagonistic zygotic drive is feasible, likely to be widespread in nature, and that it can promote a genetic “arms race” between the homo- and heteromorphic sex chromosomes. This new category of genomic conflict has the potential to strongly influence other fundamental evolutionary processes, such as speciation and the degeneration of the Y and W sex chromosomes. It also fosters a new genetic hypothesis for the evolution of enigmatic fitness-reducing traits like the high frequency of spontaneous abortion, sterility, and homosexuality observed in humans.

## Author Summary

Our study describes a new form of sexual genomic conflict that operates through the process of antagonistic green-beard effects. Although past theoretical and empirical work indicated that green-beard effects rarely operate in nature, our new theory shows why this conclusion may have to be reevaluated. We integrate modeling analysis with extant empirical work to show that the unique properties of sex chromosomes can lead to a previously unappreciated form of sexual conflict (sexually antagonistic zygotic drive) that may be widespread in nature. It operates through harmful epigenetic parental effects, asymmetrical allocation of parental investment to sons and daughters, and asymmetrical interactions between brothers and sisters. Sexually antagonistic zygotic drive is functionally analogous to meiotic drive except that it operates due to competition among opposite-sex siblings rather than between competing gametes.

## Introduction

Sex chromosomes are unusual compared to the autosomes for three reasons. First, when present in the heterogametic sex, the two types of sex chromosome are transmitted to opposite sex offspring. Second, it is common for recombination to be suppressed over a part or all of their length. Third, non-recombining sex chromosomes can evolve to become far more dimorphic than autosomes. It has long been recognized that these characteristics can contribute to genetic conflict in the context of meiotic drive, but other forms of potential sex-linked genetic conflict have received relatively little attention (reviewed in [1]). Here we evaluate the potential for the special characteristics of the sex chromosomes to contribute to a meiotic-drive like process – sexually antagonistic zygotic drive (hereafter, SA-zygotic drive) – that operates due to competition among opposite-sex siblings, rather than gamete types. The phenotypes that fuel this process are sexually antagonistic green-beard effects (hereafter SA-GrBd-effects) that only operate when there is competition among siblings.

**Figure 1:**
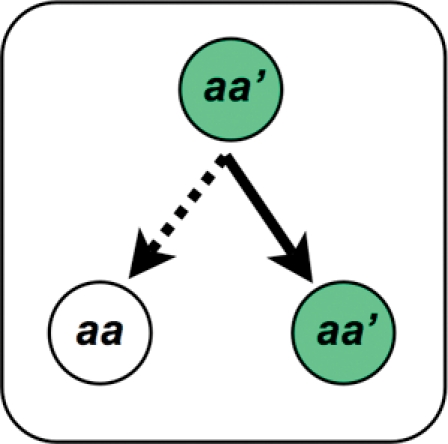
A green-beard effect mutation (a') causes its bearer to express a distinguishing phenotype (the green-beard ‘tag’ illustrated by green shading) and differentially interact with other individuals by *i)* helping other tagged individuals (increasing their survival and/or fecundity; solid arrow), and/or *ii)* harming untagged competitor individuals (dashed arrow).

A green-beard effect [2],[3] is a complex trait coded by a pleiotropic gene, or a collection of tightly linked genes, with three distinct characteristics (Figure 1): they cause the carrier to *i)* produce a distinguishing phenotype (tag), *ii)* differentiate among other individuals based on the presence or absence of the phenotype (tag-differentiation), and *iii)* augment the fitness of other individuals expressing the phenotype (tag-directed-aid). A green-beard effect is antagonistic when it reduces the competitive ability of individuals that do not express the tag, thereby increasing the fitness of individuals carrying the gene that codes for it. Because green-beard effects require complex and multifarious pleiotropy, they have previously been presumed to be rare in nature [2],[3].

However, documented examples of green-beard effects do exist (e.g., [4]–[6]). For example, in the red fire ant (*Solenopsis invicta*) egg-laying queens are heterozygotes for the *a* and *b* alleles at the *Gp-9*-locus. Homozygous queens are absent because the *b* allele is a recessive lethal and developing *aa* queens are killed by *ab* heterozygous workers (but not by *aa* homozygous workers) [4]. Queens with the *ab* genotype that were experimentally rubbed against *aa* queens were also killed by heterozygous workers. These data indicate that the *b* allele (or an allele at a tightly linked locus) displays an antagonistic green-beard phenotype because it enhances its own propagation by killing *aa* competitors (identified by their smell) that do not carry it. Green-beard effects may also feasibly operate in humans and other placental mammals (by influencing resource transfer between maternal and fetal tissue) in the context of self-recognizing gene products, e.g., homophilic cell adhesion molecules that have extracellular domains that recognize copies of themselves expressed on other cells [7]. What has not been appreciated previously, however, is that the special characteristics of sex chromosomes greatly facilitate the evolution of SA-GrBd-effects whenever there is competition among siblings.

For simplicity – but without loss of generality – we will assume male heterogamety. There are, however, some important biological differences between male (XY) and female (ZW) heterogamety, and when appropriate, we will point out how such differences may influence the course of evolution. Lastly, when we refer to the two types of sex chromosomes, we will be referring to the portion of these chromosomes that does not recombine in the heterogametic sex.

**Figure 2:**
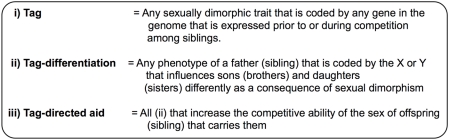
Summary of how linkage to the sex chromosomes simplifies the requisite multifarious phenotype needed to produce a sexually antagonistic green-beard effects that fuel SA-zygotic drive.

**Figure 3:**
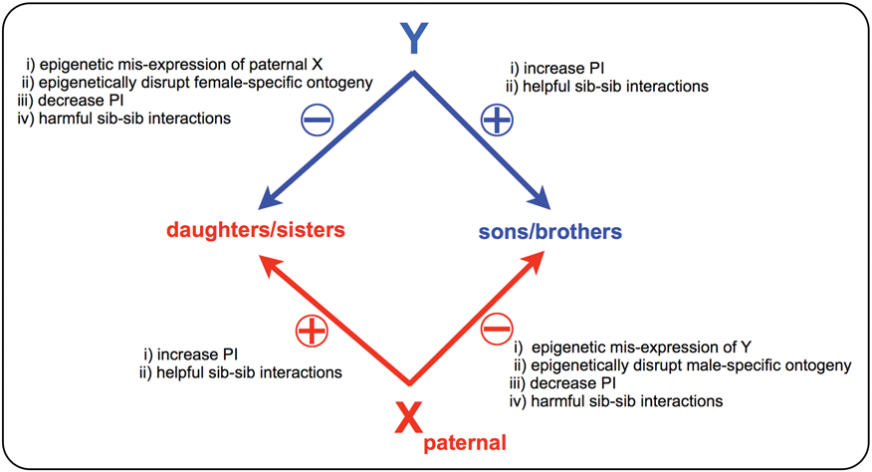
Summary of sexually antagonistic selection in males on the X and Y chromosomes.

Sex chromosomes are predicted to evolve to code for SA-GrBd-effects, and the sexually antagonistic zygotic drive that they propel, for three reasons. First, all X- and Y-linked genes co-segregate during male meiosis like a single Mendelian gene that is highly pleiotropic. As a consequence, different genes on the same sex chromosome, rather than pleiotropy of a single gene, can code for the multifarious phenotypes required for green-beard effects to operate. A second feature promoting X- and Y-coded SA-GrBd-effects is the presence of the master sex-determining gene on one of these chromosomes. This linkage creates a perfect association between the presence or absence of a father's X and Y in his offspring and all sexually dimorphic phenotypes that are coded by any gene in the genome, i.e., within a family, all daughter-specific traits are effectively paternal X-tags and all son-specific traits are effectively Y-tags (Figure 2). The final feature contributing to sex chromosomes being hot-spots for SA-GrBd-effects is competition among siblings. In this case, any X- or Y-coded phenotype that differentially influences the competitive ability of the two sexes of offspring can cause a SA-GrBd-effect in three ways (Figure 3):

1. **Epigenetic parental effects:** Defined here as any modification of an offspring's phenotype by a parent that is due to a heritable alteration of its gene expression without changing its DNA sequence, e.g., by the parent modifying the offspring's chromatin structure or by influencing the levels of steroid hormones in the embryo. A SA-GrBd-effect is produced by *i)* any X-linked gene that causes an epigenetic modification of gene expression in a father's offspring that increases the competitive ability of daughters relative to sons, and *ii)* vice versa for a Y-linked gene favoring sons.
2. **Parental investment (PI):** Defined here as any resources provided by a parent to its offspring that influences their competitive ability during sibling-sibling interactions. A SA-GrBd-effect is produced by *i)* any X-linked gene that causes fathers to be stimulated by daughter-specific traits to increase parental investment in them, and/or be stimulated to decrease parental investment in response to son-specific traits, and *ii)* vice versa for a Y-linked gene favoring sons.
3. **Competitive sib-sib interactions:** Defined here as altruistic and antagonistic interactions between siblings that influence the survival of brothers and sisters. A SA-GrBd-effect is produced by *i)* any Y-linked gene that causes brothers to be stimulated to help siblings in response to brother-specific traits and harm siblings in response to sister-specific traits, and *ii)* vice versa for a X-linked gene favoring sisters over brothers.

The same logic applies to maternal SA-GrBd-effects in the context of ZW sex determination, but the opportunity for the epigenetic modification by the mother of an offspring's gene expression may be more substantial owing to her multifarious influences on the developing egg (e.g., deposition of steroid hormones in the yolk and RNAs in the egg's cytoplasm).

The logic of SA-zygotic drive is an extension of the concepts of meiotic and gametic drive that operates postzygotically during ontogeny rather than prezygotically during meiosis and gametogenesis. As a consequence, many of the evolutionary principles developed for meiotic drive in classic papers by Sandler and Novitski (1957) [8], Hiraizumi et al. (1960) [9], Hamilton (1967)[10], Hartl (1975) [11], and others will also apply to SA-zygotic drive. However, we will show in this paper that the postzygotic operation of SA-zygotic drive (unlike the prezygotic process of meiotic drive) has a unique mode of operation that creates unprecedented, broad-scale opportunity for green-beard effects to evolve. These SA-GrBd-effects are predicted to be capable of causing a wide diversity of maladaptive phenotypes that are expressed in the diploid phase of the lifecycle.

Previous theoretical work from our laboratories has shown that linkage to the W and Z chromosomes in species with female heterogamety facilitates the evolution of selfish genetic elements that code for heritable maternal effects [12]. Here we focus predominantly on X- and Y-coded green-beard effects that evolve due to paternal epigenetic effects, parental investment (PI) by either heterogametic sex (XY or ZW), and sibling-sibling interactions (competitive sib-sib-interactions).

In the following sections we first evaluate the biological feasibility of the evolution of SA-zygotic drive of the sex chromosomes via SA-GrBd-effects, and how the autosomes would be expected to respond to such evolution. We focus especially on the feasibility of paternal epigenetic effects, because of the constraints imposed on their transmission between father and offspring via the sperm. We next develop a mathematical model of SA-zygotic drive due to coevolution between X and Y coded SA-GrBd-effects. Before discussing our collective findings, we describe how SA-zygotic drive can provides a new genetic hypothesis for the evolution of enigmatic traits, like high-frequencies of spontaneous abortion, sterility, and homosexuality, that reduce Darwinian fitness.

## Results

### Feasibility of Maternal and Paternal SA-GrBd-Effects

Consider the expression of the paternal X and Y chromosomes during spermatogenesis at a time when the developing gametes remain functionally diploid, i.e., before the primary spermatogonial cell has divided into haploid spermatids, and also while the four developing spermatids derived from each spermatagonial cell remain connected by cytoplasmic bridges that permit RNA, steroid hormones, proteins and other molecules to be exchanged (i.e., most of spermatogenesis; [13]). With sib-competition, any X-coded epigenetic modification that influences gene expression in sons, and thereby reduces their competitive ability, would be favored by genic selection. An X-linked mutation producing such a paternal epigenetic effect represents a SA-GrBd-effect between a father and his offspring because it differentially helps those offspring that carry the mutation. For example, consider an X-coded mutation that was expressed during spermatogenesis and that epigenetically modified the expression of an autosomal gene (in the zygote or developing embryo of the next generation) in a manner that disrupted a male-specific ontogenetic pathway (such as dosage compensation in *Drosophila melanogaster*) and thereby reduced the competitive ability of sons during sib-competition. In this case, the green-beard ‘tag’ is the presence or absence of the male-specific ontogenetic pathway, the ‘tag-differentiation’ is the epigenetic modification of the expression of a gene in a male-specific ontogenetic pathway that harms only (or disproportionately) sons, and the ‘tag-directed-aid’ is the resulting increased competitive ability of daughters competing with debilitated brothers. When there is sib-competition, an X-coded green-beard effect that aids (harms) one sex of offspring necessarily harms (aids) the other sex – and hence such green-beard effects are necessarily sexually antagonistic. The same logic applies to Y-coded paternal epigenetic effects that help sons by harming daughters. For example, consider a Y-coded epigenetic effect that caused mis-expression of any gene located on the paternally inherited X chromosome. This phenotype would debilitate only daughters and thereby increase the fitness of the Y chromosome when there is sib-competition. Although the Y chromosome in many species may currently contain relatively few structural genes [14], this would not have been true historically before degeneration of the Y occurred. Furthermore, a highly degenerated Y chromosome with respect to structural genes may retain substantial regulatory potential as recently shown for *D. melanogaster* [15].

With male heterogamety, sexually antagonistic epigenetic effects must operate through the sperm, which provides far more formidable barriers to expression of paternal effects compared to that of maternal effects through the egg [16]: sperm are much smaller than eggs, nearly all paternal cytoplasm is stripped away during spermatogenesis, paternal imprinting via histone modification is restricted due to protamines replacing paternal histones, and paternal imprinting via methylation is made difficult due to the nearly global demethylation of the paternal chromosomes after fertilization, as occurs in mammals. Nonetheless, a large body of extant evidence indicates that sexually antagonistic paternal (and maternal) effects can and do operate in nature, as described below.

Most research on paternal epigenetic effects in animals has focused on methylation-based imprinting in mammals. This process, however, is unlikely to contribute substantially to SA-GrBd-effects coded by the sex chromosomes because it operates through cis-acting imprinting control regions (ICRs, which are associated with relatively small proportion of genes) [17]. In contrast, the X and Y are selected to produce trans-acting gene products that epigenetically modify the expression of other parts of the genome in offspring that do not carry the coding sex chromosome.

In Text S1, we summarize extant studies to provide evidence that: *i*) epigenetic maternal and paternal effects have evolved many times that selectively kill offspring that do not carry them, *ii*) mutations that cause antagonistic parental effects that selectively harm only one sex of offspring are well documented, at least in *D. melanogaster* in the context of maternal effects, *iii*) the expression levels of hundreds of genes in *D. melanogaster* are influenced by both maternal and paternal effects, with no evidence that this phenomenon is caused by imprinting-based parent-of origin effects *iv*) trans-acting epigenetic paternal effects (that are not parent-of-origin effects, and that influence offspring that do not carry the coding gene) can be produced by RNAs produced during spermatogenesis and transferred to the zygote (as RNA or cDNA), and *v*) epigenetic maternal effects that influence the competitive ability of one sex of offspring over the other can be produced by varying steroid levels in the yolk. Collectively these studies provide evidence that X and Y-coded (and Z and W-coded) SA-GrBd-effects can feasibly evolve through both paternal and maternal effects.

Here, we briefly overview some examples of the material covered in Text S1. Antagonistic maternal effects are well documented. In mice (*HSR*, *scat^+^*, *Om* ^DDK^) and beetles (*Medea* factors), there are polymorphic alleles in natural populations that produce maternal effects that kill all of the siblings in a brood that do not carry them (reviewed in [1]). In *D. melanogaster*, there are at least three established loci that can mutate to alleles that kill sons via a maternal effect (*snl*, *sok-1*, *and sok-2*) and three that similarly kill only daughters (*l(2)mat*, *da*, and *Ne*) [18]. In birds, a maternal effect (elevated yolk androgen concentrations in the barn swallow, *Hirundo rustica*) causes enhanced growth rate of sons but reduced growth rate of daughters [19]. Trans-generational epigenetic paternal effects are also well documented. In *Caenorhabditis elegans*, a pair of tightly linked genes (*peel-1 and zeel-1*) code for a paternal effect that kills offspring that do not carry them [20]. In mice, a trans-generational epigenetic paternal effect, coded by an allele at the *Kit* locus, has been demonstrated to be mediated by RNAs produced during spermatogenesis and transmitted to the egg [21]. Human sperm transfer over 4,000 different types of RNA transcripts to the egg, including at least 68 miRNAs [16]. These studies demonstrate that mutations causing the phenotypes needed for SA-zygotic drive to operate do in fact occur.

Past evolution of antagonistic X- and Y-coded SA-GrBd-effects should have selected for adaptations by the affected sex chromosome to suppress them, and by the autosomes to suppress them whenever they harm one sex of offspring more than they help the other sex. A candidate phenotype for such suppression is the enigmatic early-inactivation of sex chromosomes (but not the autosomes) during the process of spermatogenesis. This is a well documented phenomenon in organisms as diverse as fruit flies, worms and mammals, but its adaptive significance is poorly understood [22],[23]. All chromosomes are inactivated during the latter stages of spermatogenesis when the sperm's DNA becomes highly condensed. However, the X and Y chromosomes are inactivated far in advance of the autosomes, during the early stages of spermatogenesis [24],[25]. Although the selective factors that led to the evolution of the early-inactivation of the sex chromosomes are unknown, the phenomenon is consistent with what would be expected if X and Y-coded SA-GrBd-effects have been important historically. If early-inactivation of the X and Y reduced the production of RNAs coded by these chromosomes during spermatogenesis, this would interfere with RNA-based epigenetic modification of genes in the developing sperm as well as the embryo (see Text S1). It may also protect these chromosomes from SA-GrBd-effects coded by the other sex chromosome by restricting access of gene products that modify chromatin structure (e.g., acetylation of histones). Early inactivation, however, does not completely preclude X and Y-coded SA-GrBd-effects from occurring. Recent studies indicate that ∼10% of genes on the X remain active throughout spermatogenesis in mice, and that some early inactivated X-linked genes regain activity during the latter stages of spermatogenesis [25]. Lastly, although early inactivation of the sex chromosomes might feasibly have evolved as a defense against SA-zygotic drive, meiotic drive of the sex chromosomes [26] and sex-linked sexually antagonistic alleles [23] would also select for this phenotype.

In sum, there is manifest evidence that sex chromosomes have the potential to evolve to code for SA-GrBd-effects that are mediated by parental epigenetic effects. Although the potential for such effects is greater through the egg in the case of female heterogamety, there is also substantial evidence that epigenetic paternal effects through the sperm also may be an important source of SA-GrBd-effects (Text S1). Antagonistic X and Y-coded SA-GrBd-effects may have been especially prominent during the initial stages of sex chromosome evolution, before early-inactivation of the sex chromosomes during spermatogenesis had evolved.

### Feasibility of SA-GrBd-Effects via PI in Offspring

Parental investment (PI) in offspring can be elicited by specific signals from the offspring, such as vocalizations, begging behavior, or markings such as those associated with the gaping mouth of soliciting offspring [27]. Consider an X-linked mutation that causes a father to *i)* respond to a daughter-specific trait in a manner that increased PI, or *ii)* respond to a son-specific trait that in a manner that reduced PI. Such a mutation would be favored by genic selection because it would increase the probability of its own propagation even if the net fitness of the father declined owing to the reduction in the fitness of his sons [2],[3]. The same logic applies to a Y-linked mutation that increased PI allocated to sons at the expense of daughters. The potential for such sex-specific allocation of PI is illustrated by the barn swallow (*Hirundo rustica*), in which the begging vocalizations are distinct between sons and daughters [28], and the American kestrel (*Falco sparverius*), in which the male and female nestlings have markedly different plumage [29]. In red deer, females permit sons to suckle longer and more frequently compared to daughters [30], and such sex-specific discrepancies in parental investment are well documented across a wide diversity of taxa [31]. Both solicitation displays by offspring and response to them by parents have been shown to have measurable heritability across a wide diversity of taxa, and solicitation displays are known to be influenced by maternal effects [32]. Collectively these observations indicate that there is substantial evolutionary scope for sex chromosome-coded genes to evolve that cause parents to preferentially invest in one sex of offspring at the expense of the other sex, and hence to code for SA-GrBd-effects.

### Feasibility of SA-GrBd-Effects via Competitive Sib-Sib-Interactions

The logic for sex-linked SA-GrBd-effects that are mediated by competitive sib-sib-interactions is similar to that described above for parental investment (PI). The Y is selected to promote the competitive ability of brothers, the paternal X is selected to promote the competitive ability of sisters, and the maternal X and autosomes are selected to promote the survival of the brood as a whole. In other words, these chromosomes are selected in offspring in the same way that they are selected in their parents. There is a large body of empirical evidence indicating that siblings interact differently with each other in response to the sex of the interacting partners (e.g., [33],[34], so the requisite phenotypic variation is well established for the evolution of sex-linked SA-GrBd-effects that are mediated by competitive sib-sib-interactions. Evidence that SA-GrBd-effects have actually evolved would be established by showing that there are Y-linked genes that cause males to augment the survival of brothers at the expense of sisters, and vice versa for X-linked genes.

To illustrate how easily SA-GrBd-effects could evolve via competitive sib-sib-interactions consider facultative siblicide (i.e., siblings are killed by other siblings in some, but not all, broods), which occurs in many species of birds, and some mammals [33],[35]. If an X-linked gene caused its bearer to be less stimulated to kill a sister compared to a brother (because sister-specific traits were less stimulating in inducing siblicide compared to brother-specific traits), an antagonistic green-beard effect would be manifest. As another example, cannibalism is common in a wide diversity of species during juvenile development [36],[37]. If an X-lined gene caused females to be less likely to cannibalize their sisters and/or more likely to cannibalize their brothers, such a gene would necessarily produce a SA-GrBd-effect. The same logic applies to Y-coded genes that favor brothers over sisters. More generally, any gene located on the sex chromosomes that caused a sibling to be more, or less, stimulated to be aggressive or altruistic in response to sex-specific traits of competing siblings can feasibly lead to a SA-green-beard effect.

### Selection on the Autosomes

The accumulation of X- and Y-coded SA-GrBd-effects will sometimes lead to selection pressure on the autosomes to evolve counter-measures that rescue the affected sex from the antagonistic paternal effects. If an X- or Y-coded paternal effect increases the fitness of one sex of offspring more than it harms the other sex, then the autosomes receive a net benefit and they are not selected to block the antagonistic paternal effect. Selection to block Y- and X-coded antagonistic paternal effects will occur, however, whenever they reduce the average fitness of a brood (across both sexes), and hence reduce the fitness of the autosomes. However, unlike the strong selection on the X and Y to produce, and protect themselves from, sexually antagonistic paternal effects, selection on the autosomes to block them is relatively weak. To illustrate why, consider a new Y-linked mutation coding for a paternal effect that reduces the vigor of daughters and thereby increased the juvenile competitive ability of sons. Let the fitness gain to sons (or the Y) be a positive increment (*s_son_*) and the fitness loss to daughters (or the X) be a negative increment (*s_daughter_*). The fitness effect on the autosomes is the average of *s_son_* and *s_daughter_*. Since one *s*-value is positive and the other negative, they tend to be counterbalancing, so that selection on the autosomes to block harmful paternal effects is closer to zero than selection on either the X or the Y to produce them. Hence selection on the autosomes to block antagonistic paternal effects coded by the sex chromosomes is absent, when they increase the average fitness of a brood, or relatively weak, unless they were to lead to a strong, population-wide imbalance in the sex ratio (see [38] for constraints on selection in response to a biased sex ratio). Nonetheless, there is a large number of autosomal loci that may be capable of mutating to modifiers that shut down SA-zygotic drive. As a consequence, more extreme forms of SA-zygotic drive (that reduce net brood fitness) may be eventually silenced by counter-evolution on the autosomes, or to operate episodically when new forms of SA-zygotic drive evolve that are resistant to extant autosomal modifiers (see for example [39] and references in [1], chapter 3). The same logic applies to sex-linked SA-GrBd-effects mediated by PI and competitive sib-sib-interactions.

### Modeling SA-Zygotic Drive

**Figure 4:**
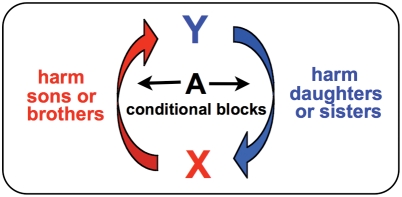
Antagonistic coevolution between the X and Y leading to recurrent episodes of SA-zygotic drive. The autosomes will only evolve to block harmful phenotypes coded by the X and Y when SA-zygotic drive causes the average fitness of the brood to decline.

If a SA-GrBd-effect evolved that was coded by the Y and that favored sons at the expense of daughters, there would be counter-selection on the X to ameliorate this effect, and vice versa if a SA-GrBd-effect evolved that was coded by the X favoring daughters. Such selection and counter-selection could potentially lead to a genetic arms race (Figure 4) with the autosomes being selected to block X- and Y-coded antagonistic paternal effects only when the net fitness of the brood was reduced. Here we explore the fate of mutations located on the X and Y chromosome that code for *i)* paternal investment (PI) that is skewed toward the sex of offspring that carries them, *ii)* epigenetic paternal effects that interfere with the ontogeny of the sex of offspring that do not carry them (and thereby reduce their competitive ability during sibling competition), and *iii)* competitive sib-sib-interactions that reduce the competitive ability of the sex that does not carry them (by helping same sex siblings or harming opposite sex siblings).

We specifically model coevolution between the X and Y. Because the X and Y do not recombine with each other, we model them as alleles at a simple Mendelian locus that determines sex (XY is male, XX is female) and pleiotropically influences epigenetic parental effects, paternal PI, or competitive sib-sib-interactions. This simplification ignores the recombination that is possible between X chromosomes in females that will lead to reduced Hill-Robertson interference on the X compared to the Y. As a consequence, our model will somewhat underestimate the rate of adaptive evolution of the X. Our model also ignores any counter-evolution by the autosomes, but this simplification should not change our qualitative conclusions owing to the expected weaker selection on the autosomes (see above section).

We start by formulating a model of differential paternal investment in sons and daughters which we then study numerically. At the end of this section, we show that a similar approach can be used, and similar conclusions apply, for epigenetic parental effects and competitive sib-sib-interactions that harm the sex that does not carry them.

#### Differential paternal investment

In our modeling analysis we trace the fate of recurrent mutations on the Y chromosome that bias paternal investment toward sons and mutations on the X chromosome that bias paternal investment toward daughters. Different mutations have variable effect sizes (drawn from a Gaussian distribution) and occur at a rate *μ* per chromosome per generation. The net effect of all mutations that have accumulated on the Y chromosome is denoted by *y* and all those that have accumulated on the X chromosome as *x*. The bias (*b*) in parental investment by an individual father toward his sons and daughters is defined by *b_son_* = *y*−*x* and *b_daughter_* = *x−y* = −*b_son_*. Fitness consequences for sons and daughters of biased paternal investment are modeled to increase with increasing bias scaled by the parameter “alpha” that controls the strength of selection (see the *Models* section below). To analyze the evolutionary dynamics in this model, we use stochastic, individual-based simulations allowing for the effects of random genetic drift, mutation, and selection. Further details of the model and simulations are given in the *Models* section below

**Figure 5:**
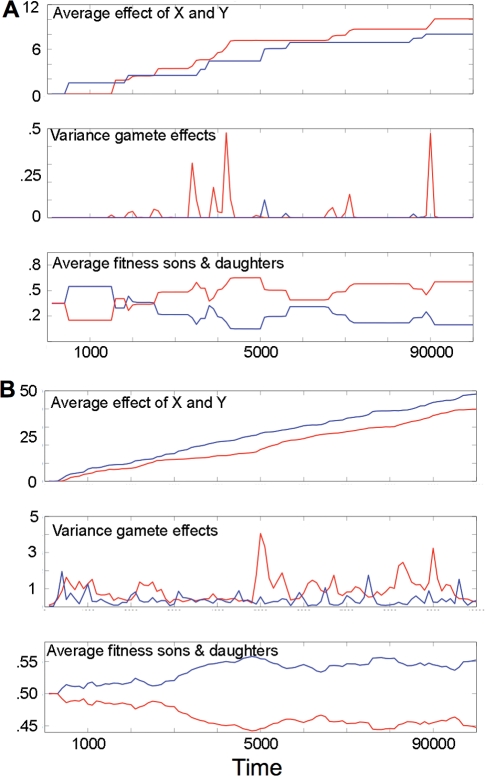
The dynamics of the average values *x* and *y*, variances of *x* and *y*, and the average fitness of sons and daughters. (A) A run with α = 0.4 and μ = 0.00001. (B) A run with α = 0.025 and μ = 0.001. Red depicts *x* and daughters and blue depicts *y* and sons.

Numerical simulations always show escalation of paternal effects *x* and *y*, as mutations on the X and Y giving advantage to daughters and sons, respectively, sweep through the population (see Figure 5). Depending on parameter values, the average effects *x* and *y* change in a more or less stepwise (see Figure 5A) or continuous fashion (see Figure 5B). Genetic variances *V_x_* and *V_y_* can be very low except for during relatively short periods of time when a new mutation goes through intermediate frequencies (see Figure 5A) or can be maintained at relatively high values (see Figure 5B). The average fitness of sons and daughters can vary significantly with periods of higher average fitness alternating between the sexes (Figure 5A).

Sex chromosomes have well established asymmetries, such as the larger number and size of X compared to Y chromosomes, that influence their rates of evolution [40]. To investigate the consequences of these asymmetries on the dynamics of our model, we have examined the effects of varying the mutation rate and the strength of selection, and also the effect reduced overall mutation rate of Y due to a diminishing number of mutable loci – which would be the case when the Y chromosome had degenerated due to a lack of recombination. A detailed description of this work is provided in Text S2, but we summarize the results here. Low mutation rate and strong selection cause the Y chromosome to lag behind the X in an arms race since the factor limiting the rate of evolution is new mutations and the X (with three-times more copies than the Y) receives three times more new mutations. High mutation rate and weak selection cause the X chromosome to lag behind the Y in an arms race because the Y is selected every generation in males, whereas only one third of the X chromosomes are selected in males each generation. When the Y is highly degenerated, the Y chromosome lags behind the X in an arms race under all conditions simulated due to its much longer wait time for new beneficial mutations to be introduced. This last result suggests that once the Y becomes highly degenerate, SA-zygotic drive would be expected to be fueled predominantly by the X and its coevolution with the autosomes. However, the recent finding by Lemos et al. (2008) [15], that the highly degenerate Y of *D. melanogaster* influences gene expression levels of over 1,000 autosomal and X-linked genes, indicates that it is premature to assume that Y chromosomes with few structural genes are minor contributors to SA-zygotic drive.

Lastly, our simulations assumed that each family was produced without cuckoldry (i.e., no departures from monogamy) and hence all offspring in a father's family were full sibs and sired by him. When departures from strict monogamy result in broods containing less than full sibs, then the strength of selection favoring SA-zygotic drive declines. Nonetheless, some level of selection for SA-zygotic drive remains so long as some offspring in a brood share the same father. We carried out an additional set of simulations in which the probability of an unrelated offspring residing in a father's brood was *ρ* (0≤*ρ*≤1) (data not shown). We found that the rate of antagonistic coevolution between the X and Y was only weakly affected when *ρ* = 0.1, but that it slowed by nearly half when *ρ* = 0.5. These simulations indicate that SA-zygotic drive is slowed but not stopped by even strong departures from monogamy.

#### Differential paternal epigenetic-effects

The above numerical results can also be applied to the case of X- and Y-coded epigenetic effects that increase the competitive ability of the sex of offspring that carries them during sibling-sibling interactions. In the case of paternal epigenetic effects, one needs to interpret *x* as the deleterious epigenetic effect of an X-linked gene on the “competitive ability” of sons in their competition with sisters. Correspondingly, *y* is interpreted as the deleterious epigenetic effect of an Y-linked gene on the “competitive ability” of daughters in their competition with sons. Then *b_son_* = *y*−*x* is the excess in competitive ability of a brother over that of his sister. Assuming that w(*b*) and w(−*b*) are fitnesses of a brother and a sister for a symmetric function *w*(.), the average fitness of sons and daughters of fathers with effects (*x*, *y*) are given by equation (1) (see *Models* section below) as before. All conclusions from the model of differential paternal investment apply immediately.

#### Differential competitive sib-sib-interactions

The relatedness between a focal male sibling's Y chromosomes, a focal female sibling's paternal X chromosome, and their brothers and sisters, is the same as that between a father's X and Y and his sons and daughters. As a result, a model of selection on the paternal X of sisters and the Y of brothers concerning their influences on brothers and sisters is qualitatively the same as that between a father and his offspring in the context of PI.

### Application of SA-Zygotic Drive to Enigmatic Traits That Reduce Fitness

**Figure 6:**
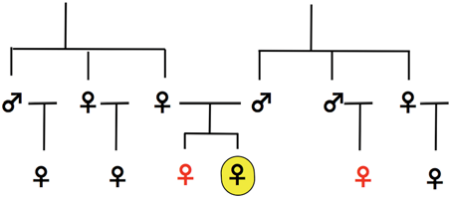
A pedigree analysis of female homosexuality. The focal homosexual individual is highlighted in yellow. Relatives expressing elevated rates of homosexuality are shown in red (based on Table 6 of Pattatucci and Hamer (1995) [41]).

SA-zygotic drive provides a previously unexplored genetic model for the evolution of traits, such as sterility and homosexuality, which reduce Darwinian fitness, but yet can attain appreciable frequency in natural populations. We illustrate the heuristic potential of the concept of SA-zygotic drive by applying this genetic model to the unusual distribution of female homosexuality in human pedigrees (Figure 6, drawn from the data presented in Table 6 of [41]). We do not claim that this phenotype represents an established example of SA-zygotic drive, only that SA-zygotic drive provides a new functional form of hypothesis that can be tested to account for this – and other enigmatic – phenotypes that presently have no other genetic explanation.

Relative to a proband (i.e., a focal homosexual female), female homosexuality was observed at rates elevated above the background level on the paternal but not the maternal side of the family, and here only among the daughters of the fathers' brothers. A proband's sisters also had elevated rates of homosexuality. There was also some indication that probands' daughters may have had elevated levels of homosexuality, but the number of daughters assayed was small, and their elevated rate of homosexuality was not statistically significant when high stringency in identifying homosexual probands was applied.

The major pattern of female homosexuality in the pedigrees was that its occurrence was elevated only in relatives (sisters and paternal female cousins) whose fathers shared the same Y chromosome, and many of the same X-linked alleles. The observation that paternal aunts did not show elevated rates of homosexuality indicates that it was the X/Y combination of the father, rather than the Y alone, that was associated with an increased probability of female homosexuality. The weaker evidence for elevated rates of homosexuality in probands' daughters is also consistent with an epigenetic effect of the sex chromosomes since paternal epigenetic effects are know to sometimes carry-over to more than one generation (e.g., see description of the *Kit*-locus in Text S1).

The association of female homosexuality with only the patriline is consistent with the operation of SA-zygotic drive, yet we are aware of no previously available genetic model that predicts this association [42]. Male homosexuality has been found to be associated with the matriline, at least in some ethnic groups (e.g., [43], but see [44]) and more recent evidence indicates that it may be caused, in part, by sexually antagonistic alleles [45]. SA-zygotic drive provides a testable hypothesis for the association of female homosexuality with a different form of genomic conflict: SA-GrBd-effects.

We see no rationale for why the Y would directly be selected to cause female homosexuality. Nonetheless, the Y is selected to epigenetically disrupt daughter-specific developmental pathways that influence their vigor. These effects could feasibly influence female sexual development outside the context of vigor through pleiotropy and lead to female homosexuality, despite there being no direct selection for this specific phenotype.

SA-zygotic drive is also predicted to influence other enigmatic fitness-reducing traits that are controlled by sex-specific processes, like the high levels in humans of both sterility (e.g., ∼10% of couples are infertile, with males accounting for 30–50% of this value [46]) and spontaneous abortion (e.g., ∼70% of human conceptions spontaneously abort, [47], most of which are not due to aneuploidy [48]). The logic in these cases is identical to that described above for female homosexuality, but in this case the disrupted sex-specific developmental pathways lead to sterility and inviability of embryos rather than homosexuality. These examples illustrate how SA-zygotic drive provides a new theoretical framework that can be used to construct a more complete set of alternative genetic hypotheses when evaluating the evolution of traits that reduce Darwinian fitness.

## Discussion

Transmission asymmetries are the biological foundation for many forms of genetic conflict. For example, the mitochondria – and cytoplasmic endosymbionts like *Wolbachia* – are typically propagated across multiple generations only through the female line of descent (matriline). Transmission of these genomes through sons (patriline) is therefore an evolutionary dead-end, as is transmission through pollen in plants. In response to this transmission asymmetry between sons and daughters, the cytoplasmically transmitted genomes of some species have evolved to kill sons or eliminate pollen production (reviewed in [1],[49],[50]). The killing of male offspring by cytoplasmically transmitted genomes is most strongly favored by natural selection when there is sib-competition because removing sons from a brood increases the availability of resources for their sisters – thereby improving the propagation of the matriline. Here we have shown that the same logic can be extended to the asymmetrical transmission of sex chromosomes to sons and daughters – leading to the hypothesis of SA-zygotic drive.

The operation of SA-zygotic drive via epigenetic parental effects has two prerequisites: *i)* sibling competition and *ii)* parental-effect mutations that harm only one sex of offspring. The first prerequisite is well established in a wide diversity of taxa (reviewed in [33]). The second prerequisite is well established in *D. melanogaster* and birds, at least for the context of maternal effects (e.g., [18],[19], see Text S1), and the recent finding of autosomal zygotic drive in *C. elegans* [20] (see Text S1) makes it clear that the requisite genetic variation is feasible via paternal effects as well. This extant empirical information, when coupled with our modeling analysis, indicates that SA-zygotic drive via epigenetic parental effects almost certainly occurs in nature, and that antagonistic green-beard effects may be more evolutionarily important than indicated by their rare demonstration in other contexts from past studies (e.g., [4]–[6]). What remains to be established is its evolutionary scope.

SA-zygotic drive via PI and sex-specific competitive sib-sib-interactions is, in principle, simpler to evolve because it does not require trans-generational epigenetic effects. There is clear evidence that sex can strongly influence both PI and competitive sib-sib-interactions (as described in detail above), so the phenotypic traits needed to fuel SA-zygotic drive are clearly in place. Nonetheless, the operation of SA-zygotic drive via sexually antagonistic competitive sib-sib-interactions and PI remains to be explored empirically, and we hope that our study will foster the relevant research.

As described in the introduction, SA-zygotic drive via SA-GrBd-effects is an extension of the logic behind meiotic drive that acts at the diploid zygote and postzygotic stages. The evolutionary scope for SA-zygotic drive may, however, far surpass that of meiotic drive, and also that of autosomal-zygotic drive (e.g., [51]) and gestational drive [7]. In male meiotic drive, selfish elements accumulate because they kill or debilitate competitor sperm that do not carry them. In female meiotic drive, driving elements accumulate when they are less prone to being transported to polar bodies because the cell's molecular motors differentiate between the centromeres of the two homologous chromosomes. Because the dimorphism between sperm carrying different chromosomes (or between the centromeres of homologs in oocytes) is relatively small, there is restricted opportunity for meiotic drive elements to distinguish between them. The small effect that a sperm's haploid genome can have on its structure and function is illustrated by *D. melanogaster* in which sperm carrying <1% of the genome (only a single “dot” chromosome 4) are fully functional [52]. Similarly, there is relatively little dimorphism between zygotes and embryos that do and do not carry a genetic element, such as a *Medea* factor [51], that causes autosomal-zygotic drive, or between fetuses expressing different self-recognizing alleles hypothesized to mediate gestational drive [7]. In sharp contrast, there are many sexual dimorphisms (and the ontogenetic pathways that produce them) that distinguish male and female offspring. These numerous dimorphic phenotypes are expected to substantially increase the evolutionary scope for sex-linked, SA-zygotic drive to operate, since any one of them, irrespective of the genes coding for them, represents a phenotypic “tag” for a SA-GrBd-effect. In addition, sex-specific PI and sib-sib interactions, which are well documented in nature (see above), as well as epigenetic modification of any sex-specific phenotype (also well established in nature, see Text S1), can readily produce both “tag differentiation” and “tag-directed aid” whenever these phenotypes are coded by the sex chromosomes and there is competition among siblings. Therefore, SA-zygotic drive has the potential to be a far more pervasive process than meiotic drive, gestational drive, and autosomal-zygotic drive.

The accumulation of Z- and Y-linked mutations that reduce the competitive ability of daughters, or W- and X-linked mutations that reduce the competitive ability of sons, would be expected to create counter-selection on the opposite sex chromosome (and sometimes the autosomes) to rescue the affected sex from harm, and thereby potentially lead to a genetic arms race. If such an arms race occurred, it would contribute to *i)* rapid genetic divergence between allopatric lineages – thereby potentially contributing to the evolution of postzygotic reproductive isolation during the process of speciation, *ii)* the decay of the nonrecombining sex chromosome via genetic hitchhiking, and *iii)* the evolution of elevated levels of sterility, embryo inviability, and homesexuality that exceed what would be expected by mutation-selection balance.

Each time a new SA-GrBd-effect mutation is recruited to the nonrecombining W or Y chromosome, one or more mildly deleterious mutations can accumulate on this chromosome due to genetic hitchhiking (hitchhiking-decay, [53]–[55]). If there is a substantial pool of SA-GrBd-effect mutations that can potentially accumulate on nascent W or Y chromosomes, then coevolution between the W or Y and the rest of the genome could be a powerful process driving their decay.

Antagonistic coevolution between X and Y-coded SA-GrBd-effects, and sometimes including their autosomal suppressors, would be expected to cause otherwise conserved genes to evolve rapidly. The consequent genetic divergence between allopatric populations could be a potent factor leading to Dobzhansky-Muller incompatibilities [56],[57]. In accordance, recent evidence indicates the sex chromosomes are coding hotspots for Dobzhansky-Muller incompatibilities in *Drosophila* [58]. SA-zygotic drive also provides an unexplored genetic route to the evolution high frequencies of fitness-reducing traits like sterility and homosexuality due to its predicted disruption to sex-specific ontogenetic pathways, as described above.

If SA-zygotic drive can so readily evolve, then why has it not already been widely reported, as has meiotic drive? One explanation is that early inactivation of the sex chromosomes during gametogenesis has largely shut down SA-zygotic drive in most species with ancient X and Y sex chromosomes, which included most multicellular model organisms. However, this same logic would apply to sex-linked meiotic drive, which has been observed in model organisms like *Drosophila*. Another explanation is that SA-zygotic drive has been misidentified as meiotic drive in non-model organisms that have not been analyzed genetically. A more satisfying explanation, however, is that most SA-zygotic drive may not have the strong effects that would lead to easily noticeable phenotypes, such as strongly distorted brood sex ratios. Antagonistic mutations that code for parental effects that kill offspring that do not carry them (e.g., *Medea* in *Tribolium* and *peel-1/zeel-1* in *C. elegans*, which have only recently been discovered) may metaphorically represent the tip of an iceberg of a larger number of potential SA-GrBd-effects that have smaller effects, and therefore would not be detected unless specifically looked for with large sampling effort.

Two lines of evidence suggest that SA-zygotic drive would typically not produce an easily observable lethal phenotype. First, most sperm-mediated trans-generational epigenetic effects (other than methylation-based imprinting, which is not expect to fuel SA-zygotic drive, as described earlier) that have been studied to date do not fully silence their target genes (i.e., they act more like rheostats than on/off switches; reviewed in [59]). Second, even if the target gene of an epigenetic modification were silenced, the vast majority of loss of function mutations are not homozygous-, hemizygous, nor heterozygous-lethal (e.g., as established in *Drosophila*; [60]–[62]), although it is common for non-lethal mutations in *Drosophila* (and lethal mutations in the heterozygous state) to reduce the juvenile competitive ability of their carriers (reviewed in [63]). For these reasons, trans-generational epigenetic effects that cause reduced competitive ability (but not unconditional lethality) of the sex of offspring that does not carry them, are expected to play the predominant role in coding for SA-zygotic drive.

Is SA-zygotic drive expected to be a common, but overlooked, phenomenon? We have provided what we think is convincing evidence that SA-zygotic drive, fueled by SA-GrBd-effects, is a plausible evolutionary process because the requisite phenotypes for its operation are known to occur. It is a more difficult matter, however, to predict how commonly this phenomenon is likely to be manifest in nature. Large, easily observed SA-GrBd-effects (that harm one sex more than they help the other, e.g., son- or daughter-killers), would select for suppressors on the autosomes. In this case, the numerical excess of autosomal compared to sex-linked genes should lead to autosomal silencing of this form of SA-zygotic drive, or at least make it episodic. However, less extreme forms of SA-zygotic drive are not predicted to be opposed by the autosomes, as described above, so this – more difficult to discern – form of SA-zygotic drive is predicted to be most common. In this case the prevalence of SA-zygotic drive will depend only on the mutation rate to alleles coding for small SA-GrBd-effects – a parameter that is presently unknown.

Throughout this manuscript we have emphasized harm, rather than altruism, as the phenotype mediating SA-zygotic drive. We have done this because we have assumed that there is competition among siblings for limiting resources. In this case, any phenotype that aids one sex of offspring in a family will make this sex more competitive, and thereby harm the opposite sex. Thus, helping one sex in a brood will necessarily harm the other sex. We also have focused predominantly on the sex chromosomes themselves. However, in some cases the mitochondria and other cytoplasmic genomes will co-segregate with a sex chromosome (e.g., the W sex chromosome in species with female heterogamety co-segregates with all cytoplasmically transmitted genomes). In this case, SA-zygotic drive also may be influenced by phenotypes coded by the cytoplasmic genomes that co-segregating with the sex chromosomes.

### Predictions

Our theory of SA-zygotic drive can be used to generate testable predictions. The major – and counterintuitive – prediction concerning SA-zygotic drive is that a father's Y chromosome will be observed to sometimes strongly influence the fitness of his daughters and his X will similarly influence his sons. In the case of female heterogamety, analogous predictions apply to the W and Z chromosomes. The empirical work described above (e.g., the *Kit^tm1alf^* mutation in mice [21] and the sex-specific maternal effect mutations in *D. melanogaster* [18] and birds e.g., [19] proves that these types of effects can feasibly evolve (see Text S1). It has also been established in inbred strains of mice that a father's Y chromosome can influence the behavior [64] and immune function [65] of his daughters. Our theoretical study provides a motivation for researchers to screen in future studies for an influence of the X and Y (and W and Z) on the sex of offspring that does not carry them.

A second prediction is that heritable paternal effects on offspring fitness should be found to be more common, and larger in magnitude, in species with male heterogamety, and within this group this pattern should be strengthened as the degree of monandry and sib-sib interactions increase. The absence of strong paternal effects in species lacking male parental care is commonly assumed in studies of quantitative genetics. Our theoretical work, however, predicts that this assumption will sometimes be violated due to polymorphism (sex linked or autosomal) influencing the expression of paternal SA-GrBd-effects.

A taxonomic prediction is that SA-zygotic drive should be especially prevalent in birds. This taxon has unusually high levels of monogamy (within a breeding season and despite low levels of extra-pair fertilizations, [66], an absence of inactivation of the W and Z sex chromosomes during oogenesis [23], and high levels of parental care and sib-sib interactions. Birds also have female heterogamety which facilitates parental epigenetic effects through the mother's large contribution to the embryo of RNAs and steroid hormones. The combination of these characteristics makes birds an ideal taxon to test for the existence of SA-zygotic drive.

The main prediction concerning competitive sib-sib-interactions is that, in species with sex chromosomes, same-sex sibling interactions should be more altruistic and less aggressive compared to between-sex interactions (excluding species with other factors magnifying same-sex sib competition, such as those with local mate competition or early dispersion of only one sex of offspring). A similar prediction has been made earlier by several other researchers (reviewed in [67]) based on the idea that X and Z sex chromosomes segregate the same way that haploid genomes do in species with haplodiploid sex determination. In haplodipoid species, full sisters are more closely related to each other (R = proportion of shared polymorphic alleles = ¾) than to brothers (R = 1/4), and more closely related than bothers are to each other (R = ½). As a consequence, sister-sister interactions are predicted to be the more cooperative. Assuming that the heteromorphic sex chromosome (Y or W) is too degenerate to code substantially for cooperation, the X and Z have the same relationship in brothers and sisters as whole genomes do in haplodiploids, and hence X and Z-linked genes are predicted to evolve to make members of the homogametic sex to be more cooperative with each other. There is some support for this prediction based on taxonomic comparisons. For example, long-term cooperative groups are more common among brothers in birds and sisters in mammals [68]. However, we have found no relevant information (pro or con) in the literature concerning the more specific prediction of SA-zygotic drive that during sib-competition opposite-sex individuals will be more competitive with each other compared to same-sex individuals. We suspect, however, that this information may have been collected incidentally in many studies of animal behavior – but unreported. Our study should provide an impetus to publish such comparisons.

The main prediction concerning PI is that, all else being equal, asymmetry in its allocation to sons and daughters should be higher, and sometimes more variable, in the heterogametic compared to the homogametic parent. The ‘all else being equal’ qualifier is important here because in taxa like birds males may vary in PI more than females owing to varying uncertainty in paternity. We have been unable to find any studies reporting this metric (so we have found neither positive nor negative evidence), but again we suspect that it may have been collected incidentally but unreported in past studies of animal behavior.

Lastly, when there is sib-competition, sexual dimorphism of offspring is predicted to be reduced in species with sex chromosomes, and within this group, lower yet when there is PI from the heterogametic parent. To illustrate the rationale for this prediction, suppose that an X-coded paternal effect evolved that caused fathers to increased PI in response to a daughter-specific trait, or reduce PI in response to a son-specific trait. Sons would be selected to converge in phenotype with their sisters, leading to the evolution of reduced sexual dimorphism during the period of sib-competition.

### Conclusions

Nonrecombining sex chromosomes create an unappreciated opportunity for the evolution of zygotic drive via sexually antagonistic green-beard effects whenever there is competition among siblings. The evolutionary scope for SA-zygotic drive is predicted to exceed that of meiotic, gestational, and autosomal-zygotic drive because all sexually dimorphic traits can acts as “tags” for sexually antagonistic green-beard effects. These sexually antagonistic phenotypes can, in principle, lead to an arms race between the two types of sex chromosomes (sometimes also including the autosomes, which can slow, and temporarily or permanently halt, the process) that can *i)* accelerate the degeneration of the heteromorphic sex chromosome, *ii)* cause genes that would otherwise be highly conserved to diverge among allopatric lineages and thereby leading to the evolution of Dobzhansky-Muller incompatibilities during speciation, and *iii)* lead to the disruption of sex-specific ontogenetic pathways that can lead to increased levels of expression of traits, like homosexuality and sterility, that lower Darwinian fitness. We need to stress in closing, however, that we have only established the potential for SA-zygotic drive to operate in nature and it will remain a feasible but unproven possibility until suitable empirical testing has been undertaken.

## Models

For simplicity we assume that each mating results in two offspring. Let the parameter *b_son_* characterize the bias in paternal investment toward sons in families with one daughter and one son, with *b_son_* = 0, *b_son_*>0, and *b_son_*<0 implying equal investment in both offspring, higher investment in the son and higher investment in the daughter, respectively. Let *x* and *y* be the (additive) effects of X- and Y-linked genes in the father on the bias of his paternal investment. More specifically, we let *b_son_* = *y*−*x* and *b_daughter_* = *x*−*y* = −*b_son_*, so that X-linked genes favored by selection (that increase *x*) cause the father to invest more in his daughter while Y-linked genes favored by selection (that increase *y*) cause him to invest more in his son. We assume that the fitness of a brother and a sister in a brother-sister brood are *w*(*b_son_*) and *w*(*b_daughter_*) = *w*(−*b_son_*), respectively, where *w*(.) is a symmetric function changing from 0 to 1 as *b_son_* changes from −∞ to +∞ with *w*(0) = 0.5 and *w*(*b_son_*)+*w*(*b_daughter_*) = 1 (see below). Interpreting fitness as the amount of a resource available, the latter two equalities imply that the overall amount of resource is fixed (at 1) and that with no bias (i.e. if *b_son_* = *b_daughter_* = 0), both sex of offspring get an equal share (equal to 0.5). The symmetry of this relationship is motivated by the idea that an extra unit of PI given to one sex of offspring is taken away from the other sex of offspring, and this implicitly assumes that the benefit of an extra unit of PI is equal to the cost of losing a unit of PI. Finally, we assume that fitness of each offspring in the families with the same-sex of offspring is equal to 0.5. Under these conditions, the average fitness of sons and daughters of fathers with effects (x, y) are
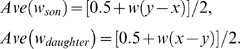
(1)The average fitness of sons and daughters given by eq. 1 are both limited to the interval [0.25, 0.75].

**Figure 7:**
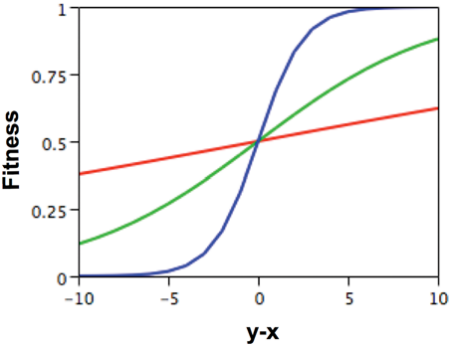
Fitness function for α = 0.4 (stronger selection, steeper blue curve), α = 0.1 (moderate selection, green curve), and α = 0.025 (weaker selection, red curve).

The evolutionary dynamics in this model were analyzed by using stochastic, individual-based simulations that allowed for the effects of random genetic drift, mutation, and selection. Generations were discrete and non-overlapping and the population size was fixed at N males and N females. Individuals entered the mating pool with probabilities proportional to *w*_son_ and *w_daughter_* for males and females, respectively, and mating was random within the mating pool. The number of matings (and families produced) per individual of each sex was a binomial random variable. Mutation occurred in both parents with probability *μ* per chromosome per generation and changed effects *x* or *y* by a random value taken from a normal distribution with a mean of zero and a standard deviation of one. The fitness function *w*(.) was specified as:

In sons: *w*(*b_son_*) = *exp*(α*b_son_*)/[*exp*(α*b_son_*)+*exp*(−α*b_son_*)] in a son/daughter family,  = 0.5 in a son/son family
In daughters: *w*(*b_daughter_*) = *exp*(α*b_daughter_*)/[*exp*(α*b_daughter_*)+*exp*(−α*b_daughter_*)] in a son/daughter family,  = 0.5 in a daughter/daughter family

where α>0 is a parameter measuring the strength of selection (larger values of *α* imply stronger selection; see Figure 7). We assumed that initially there was no genetic variation and the *x* and *y* effects of all individuals were set to zero. We varied the mutation rate *μ* and the strength of selection *α* while the number of individuals of each sex was always set at *N* = 1000. For each parameter combination, we did 20 runs each for 10000 generations. Overall, the dynamics are expected to be very similar to those observed in models of sexual conflict over mating rate [69]–[71].

## Supporting Information

Text S1 Mechanisms for the operation of SA-zygotic drive.
(0.15 MB PDF)

Text S2 The influence of varying mutation rate, strength of selection, and degeneration of the Y chromosome on coevolution between the X and Y chromosomes.
(0.39 MB PDF)
