# Supplementary material for: Sexually Antagonistic “Zygotic Drive” of the Sex Chromosomes
Source: PLoS Genet. 2008 Dec 19;4(12):e1000313. doi: 10.1371/journal.pgen.1000313 (PMC2596966; doi:10.1371/journal.pgen.1000313)
Supplement: Text S1 — Mechanisms for the operation of SA-zygotic drive. (0.15 MB PDF) [file pgen.1000313.s001.pdf]

Supporting Information

Text-1: Mechanisms for the operation of SA-zygotic drive

Antagonistic maternal effects that kill offspring that do not carry the genes that code for them are well documented. In the flower beetle (*Tribolium castaneum*) there are four independently-evolved *Medea* factors that kill offspring that do not carry them [1,2]. In mice three different elements (*HSR, scat*$^{+}$*, Om*^{DDK}) have a similar maternal effect causing the selective killing of offspring that do not carry these elements [3-6]. Direct evidence for the existence of epigenetic parental effects that target gender-specific pathways comes from studies of *D. melanogaster*. There are at least three established loci that can mutate to alleles that kill sons via a maternal effect (*snl, sok-1, and sok-2*) and three that similarly kill only daughters (*l(2)mat, da,* and *Ne*) (list compiled in [7]). These studies clearly demonstrate that the types of parental-effect mutations (that harm only one sex of offspring) needed to fuel SA-zygotic drive exist, at least in the context of maternal effects.

More subtle forms of antagonistic parental effects have also been documented that influence the two sexes of offspring differently. Recent manipulative experiments with the barn swallows (*Hirundo rustica*) showed that elevated yolk androgen concentrations led to enhanced growth rate of sons but reduced growth rate of daughters [8]. In the zebra finch (*Aeniopygia guttata*) the opposite pattern was observed [9], and in the American kestrel (*Falco sparverius*) higher androgen levels in the yolk reduced the growth rate of male, but not female, nestlings [10]. In the case of the zebra finch, the maternal-effect enhanced growth rate of daughters was associated with an increase in their begging rate. These studies demonstrate how simple it is to produce an antagonistic green-beard effect

coded by the sex chromosomes: A Z-linked mutation that, via a maternal effect, elevated yolk androgen concentration in barn swallows would be a sexually antagonistic green-beard effect, as would be a W-linked mutation with the same maternal effect in the zebra finch. But can similar, sexually antagonistic paternal effects also occur?

It is well established that some strains of the bacterial endosymbionts *Wolbachia* and *Cardinium* (those producing cytoplasmic incompatibility, *CI*) have evolve to produce a paternal effect that selectively kills zygotes that do not carry the same strain of bacteria (reviewed in [11]). Although the causative agent is an endosymbiont, this example nonetheless represents an empirical demonstration of an antagonistic paternal effect. Recent evidence indicates that antagonistic paternal effects have evolved in *C. elegans*. In this species a pair of tightly linked autosomal genes (*peel-1,* paternal effect epistatic embryonic lethal-1*, and zeel-1,* zygotic epistatic embryonic lethal-1) cause a paternal effect that kills offspring that do not carry them [12]. This lethal phenotype is only produced as a paternal effect and not as a maternal effect.

In reciprocal crosses between inbred lines, Gibson et al. (2004) [13] used microarray analysis to show that at least 289 genes in the genome of *Drosophila melanogaster* showed a pattern of gene expression in F_1 hybrid females that depended on the direction of the cross. About 40% of these genes closely matched the paternal level of expression and 60% closely matched the maternal level of expression. Wittkopp et al. (2006) [14] later assayed a small subset of the genes identified by Gibson et al. (2004) [13] using qPCR of specific alleles. They showed that the differences in expression levels between reciprocal crosses were not due to genomic imprinting of the specific alleles (i.e., an expression level of individual alleles that depended on the parent-of-origin) but

due instead to other maternal or paternal epigenetic effects influencing the levels of expression of both the alleles in an offspring. Collectively, these data indicate, at least in *D. melanogaster*, that both maternal and paternal epigenetic parental effects influencing gene expression can be substantial.

Maternal RNAs and hormones packaged in the egg and coded by the diplotype of the mother provide ample opportunity for maternal epigenetic paternal effects, but how could such paternal effects be produced via the sperm? Recent evidence indicates that RNA packaged in the sperm can mediate paternal effects. In the mouse, a dominant mutation at the *Kit* locus (Kit^{tm1alf}) produces white spots on the tail and feet of heterozygous individuals (Kit^{+}/Kit^{tm1alf}) [15]. When a heterozygote is crossed to a homozygous wild type individual, genetically wild type offspring ($\mathrm{Kit}^{+}/\ \mathrm{Kit}^{+}$) commonly express the mutant phenotype, and this epigenetic effect is sometimes transmitted to the following generation of offspring. A series of experiments demonstrated that the mutant *Kit* gene is transcribed postmeiotically during spermatogenesis, and that its mRNA is packaged in the sperm and transmitted to eggs, which through an epigenetic process leads to the expression of the mutant phenotype in offspring that do not carrying the dominant Kit^{tm1alf} allele. Microinjection into fertilized eggs of total RNA from Kit^{+}/Kit^{tm1alf} heterozygotes, or Kit-specific interfering microRNAs, also produced the mutant phenotype. These studies are proof-of-principle that the epigenetic molecular machinery is in place for paternal SA-GrBd-effects to evolve.

Recent research with sperm-mediated-gene-transfer (SMGT) also indicates that RNAs delivered by the sperm can cause epigenetic effects. In one set of experiments with mice, sperm were incubated with an RNA vector marked with a beta-galactocidase gene

(*beta-gal*). This RNA was taken up by the sperm, reverse-transcribed to DNA, transmitted to the egg during in vitro fertilization, and then mosaically propagated in both the offspring and, in some cases, to the subsequent generation. The reverse transcribed gene rarely became integrated into the mouse chromosomes, and was presumably propagated as an episome [16]. Reverse transcription of a sperm's RNAs is made possible by two pulses of reverse transcriptase activity; one during late spermatogenesis and another during the early cleavage stages of the embryo (reviewed in [17]). Human sperm transfer over 4,000 different types of RNA transcripts to the egg, including at least 68 miRNAs [18], but it is still unclear what functions, if any, they provide [19]. Another possible mechanism for paternally coded epigenetic parental effects is histone modification of the small proportion of DNA that is not protamine bound during the latter stages of spermatogenesis (e.g., ~15 % of human sperm DNA remains histone, rather than protamine, bound; [20]). Collectively these studies provide further evidence that, in principle, there is the potential for sex chromosomes to epigenetically modify unlinked genes expressed in offspring that do not carry them, and thus that X and Y-coded SA-GrBd-effects can feasibly evolve.

1. Beeman RW, Friesen KS, Denell RE (1992) Maternal-Effect Selfish Genes in Flour Beetles. Science 256: 89-92.

2. Beeman RW, Friesen KS (1999) Properties and natural occurrence of maternal-effect selfish genes ('Medea' factors) in the Red Flour Beetle, Tribolium castaneum. Heredity 82: 529-534.

3. Weichenhan D, Traut W, Kunze B, Winking H (1996) Distortion of Mendelian recovery ratio for a mouse HSR is caused by maternal and zygotic effects. Genetical Research 68: 125-&.

4. Hurst LD (1993) scat+ is a selfish gene analogous to Medea of Tribolium castaneum. Cell 75: 407-408.

5. Peters LL, Barker JE (1993) Novel inheritance of the murine severe combined anemia and thrombocytopenia (Scat) phenotype. Cell 74: 135-142.

6. Renard JP, Baldacci P, Richoux-Duranthon V, Pournin S, Babinet C (1994) A maternal factor affecting mouse blastocyst formation. Development 120: 797-802.
7. Belote JM, Lucchesi JC (1980) Male-specific lethal mutations of Drosophila melanogaster. Genetics 96: 165-186.
8. Saino N, Ferrari RP, Romano M, Martinelli R, Lacroix A, et al. (2006) Maternal allocation of androgens and antagonistic effects of yolk androgens on sons and daughters. Behavioral Ecology 17: 172-181.
9. von Engelhardt N, Carere C, Dijkstra C, Groothuis TGG (2006) Sex-specific effects of yolk testosterone on survival, begging and growth of zebra finches. Proceedings of the Royal Society B-Biological Sciences 273: 65-70.
10. Sockman KW, Weiss J, Webster MS, Talbott V, Schwabl H (2008) Sex-specific effects of yolk-androgens on growth of nestling American kestrels. Behavioral Ecology and Sociobiology 62: 617-625.
11. Burt A, Trivers R (2006) Genes in conflict : the biology of selfish genetic elements. Cambridge, Mass.: Belknap Press of Harvard University Press. viii, 602 p., [608] p. of plates p.
12. Seidel HS, Rockman MV, Kruglyak L (2008) Widespread genetic incompatibility in C. elegans maintained by balancing selection. Science 319: 589-594.
13. Gibson G, Riley-Berger R, Harshman L, Kopp A, Vacha S, et al. (2004) Extensive sex-specific nonadditivity of gene expression in Drosophila melanogaster. Genetics 167: 1791-1799.
14. Wittkopp PJ, Haerum BK, Clark AG (2006) Parent-of-origin effects on mRNA expression in Drosophila melanogaster not caused by genomic imprinting. Genetics 173: 1817-1821.
15. Rassoulzadegan M, Grandjean V, Gounon P, Vincent S, Gillot I, et al. (2006) RNA-mediated non-mendelian inheritance of an epigenetic change in the mouse. Nature 441: 469-474.
16. Smith K, Spadafora C (2005) Sperm-mediated gene transfer: applications and implications. Bioessays 27: 551-562.
17. Miller D (2007) Spermatozoal RNA as reservoir, marker and carrier of epigenetic information: Implications for cloning. Reproduction in Domestic Animals 42: 2-9.
18. Boerke A, Dieleman SJ, Gadella BM (2007) A possible role for sperm RNA in early embryo development. Theriogenology 68: S147-S155.
19. Amanai M, Brahmajosyula M, Perry AC (2006) A restricted role for sperm-borne microRNAs in mammalian fertilization. Biol Reprod 75: 877-884.
20. Krawetz SA (2005) Paternal contribution: new insights and future challenges. Nat Rev Genet 6: 633-642.
