# Supplementary material for: Sexually Antagonistic “Zygotic Drive” of the Sex Chromosomes
Source: PLoS Genet. 2008 Dec 19;4(12):e1000313. doi: 10.1371/journal.pgen.1000313 (PMC2596966; doi:10.1371/journal.pgen.1000313)
Supplement: Text S2 — The influence of varying mutation rate, strength of selection, and degeneration of the Y chromosome on coevolution between the X and Y chromosomes. (0.39 MB PDF) [file pgen.1000313.s002.pdf]

Supporting Information

Text S2: The influence of varying mutation rate, strength of selection, and degeneration of the Y chromosome on coevolution between the X and Y chromosomes

Table S1 illustrates the effects of μ (chromosome-wide mutation rate) and α (strength of selection, see *Models* section of main manuscript) on the average rate of evolution. The average rate of evolution was defined as the average (over 20 runs) of the change in the mean values of x and y over the second half the run: $(x(10000) - x(5000) + y(10000) - y(5000))/2$. As expected, stronger selection (i.e., larger α) and higher mutation rate μ increase the rate of evolution. Note that because the standard deviation of mutation effects was set to 1, the numbers in the table can roughly be interpreted as the number of "average" mutations fixed per 5000 generations. Overall, sexually antagonistic selection via paternal effects can result in very rapid genetic changes. Note also that there was a lot of variation in the rates of evolution around the average values (see the web page at http://neko.bio.utk.edu/~sergey/xy/summary.html for data on individual runs).

Table S1. The average rates of evolution (i.e., the number of average mutations fixed per 5000 generations) for different values of α and μ.

	μ=0.00001	μ=0.0001	μ=0.001
α=0.04	5.04	43.90	161.98
α=0.01	2.43	15.53	63.93
α=0.0025	0.67	4.68	24.70

Table S2 shows the effects of μ and α on the levels of genetic variation V_x and V_y maintained in x and y effects. For each run, fifty equally spaced points from the second half of the run were used (i.e., at generations 5,100; 5,200, 5,300 . . . and 10,000) giving 1000 data points (50x20) for each parameter combination. As expected, the amount of genetic variation

maintained increases with the rate of mutation. There is always more variation in x effects than in y effects (which is expected because there are three times more X chromosomes than Y chromosomes). For intermediate and large mutation rates, there is a tendency for genetic variances to increase with relaxing selection (i.e., decreasing α).

Table S2. The average levels of genetic variation $100V_x$ and $100V_y$ for different values of α and μ.

	μ=0.00001	μ=0.0001	μ=0.001
α=0.04	0/0	0.65/0	17.58/19.94
α=0.01	1.37/0	0.96/0.19	17.95/2.63
α=0.0025	0/0	4.10/0	21.56/4.47

Because of the stochastic nature of the process, there is a lot of variation not only in the rates of evolution and the levels of genetic variation, but also in fitness consequences of antagonistic coevolution. Figure S1 provides a set of histograms showing the fitness skew, i.e. the difference in the average fitness of males and females, $\overline{w}_{son} - \overline{w}_{daughter}$. For each run, five equally spaced points from the second half of the run were used (at generations 6,000; 7,000, 8,000, 9,000 and 10,000) giving 100 data points (20x5) for each parameter combination. Positive values of $\overline{w}_{son} - \overline{w}_{daughter}$ imply overall fitness advantage of sons whereas negative values of $\overline{w}_{son} - \overline{w}_{daughter}$ imply overall fitness advantage of daughters. The average fitness of sons and daughters were rarely matched and some fitness skew was almost always present. The histograms show that increasing the strength of selection results in more variation in the fitness skew. With the smallest mutation rate, the histograms appear to be symmetric around the origin so that no sex has a fitness advantage on average. With the highest mutation rate the histograms are skewed to the right implying fitness advantage of sons over daughters. One can interpret these results in the following terms. The genes on Y are subject to selection every generation

(because they always go from a father to a son). The genes on X are subject to selection only one third of the time since only one third of the X chromosomes reside in males. Therefore Y-linked genes are subject to stronger selection than X-linked genes. However, there are 3 times more X-linked genes than Y-linked genes, so the X receives 3-times more mutational input. The overall rate of evolution depends on both mutation rate and the strength of selection. With low mutation rates, X-linked genes can keep up with Y-linked genes because there is more variation in x. If mutation rates are high enough, then Y-linked genes will have the net advantage (and sons will have a fitness advantage).

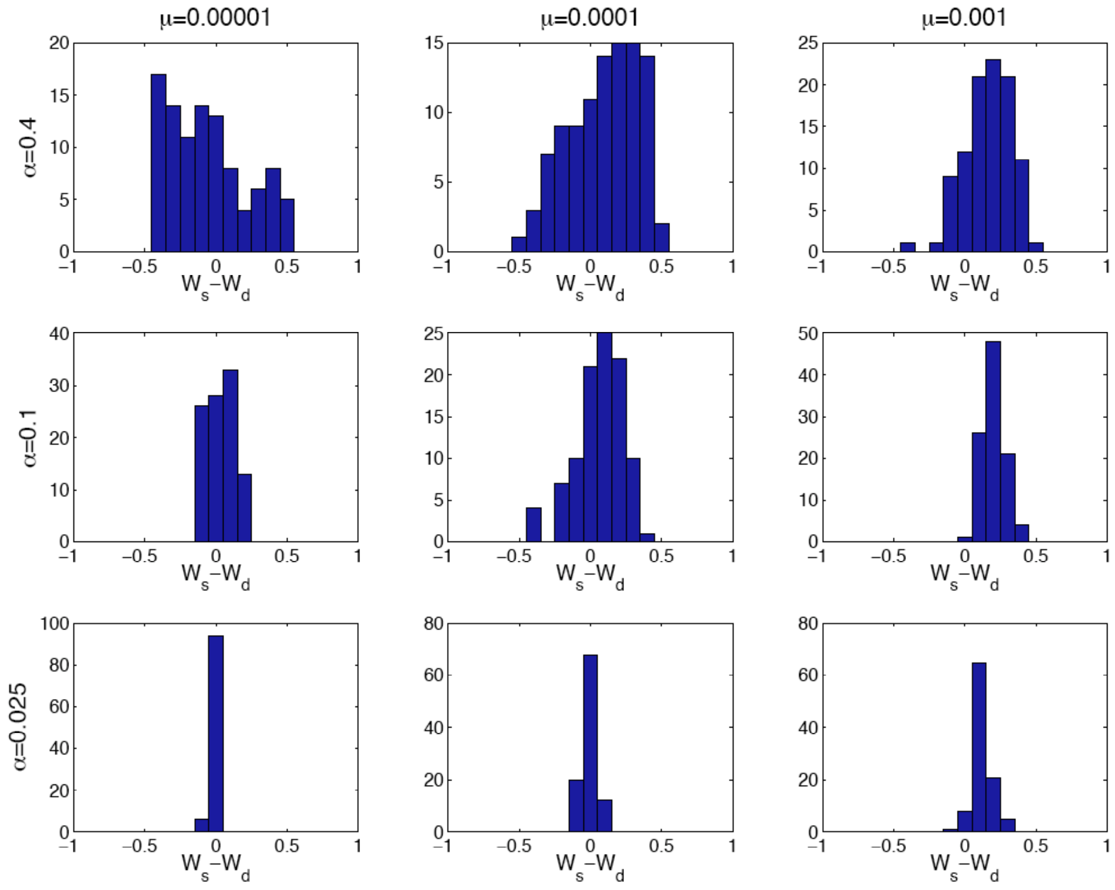

Figure S1. The effects of α and μ on fitness skew $W_x - W_y$. 100 data points for each parameter configuration.

These relationships assume that the X and Y have similar numbers of mutable sites. As the Y chromosome degenerates over evolutionary time, the X gains an evolutionary advantage over all areas of the parameter ranges of mutation rate and selection strength that we investigated (see Table S3 and Figures S2 and S3), indication that the X will typically be ahead in an arms race (and daughters will have a fitness advantage) when the Y is highly degenerate.

Table S3. The effects of a reduced mutation rate in Y on the average levels of genetic variation $100V_x$ and $100V_y$. Mutation rate in Y is one tenth of that in X. The zero entry means that the corresponding variance was smaller than 0.00001.

	μ=0.00001	μ=0.0001	μ=0.001
α=0.04	0/0	0/4.15	50.56/0
α=0.01	0/0	0/0	34.68/9.81
α=0.0025	0/0	0.23/0	5.34/1.24

Lastly, since our model assumes symmetrical, but opposite, fitness gains and losses to the two sexes of offspring, X-linked variation is effectively neutral in females in our simulations (a mutation is helping one sex of offspring by exactly the same amount that it hurts the other sex of offspring). In nature the fitness effects of sex-linked mutations causing biased PI may not be exactly offsetting. Nonetheless, so long as they are similar in magnitude and opposite in sign, selection in the homogametic sex will be weak compared to selection in the heterogametic sex so that mutations on the X and Z chromosomes will be selected nearly as strongly as those on the Y and W.

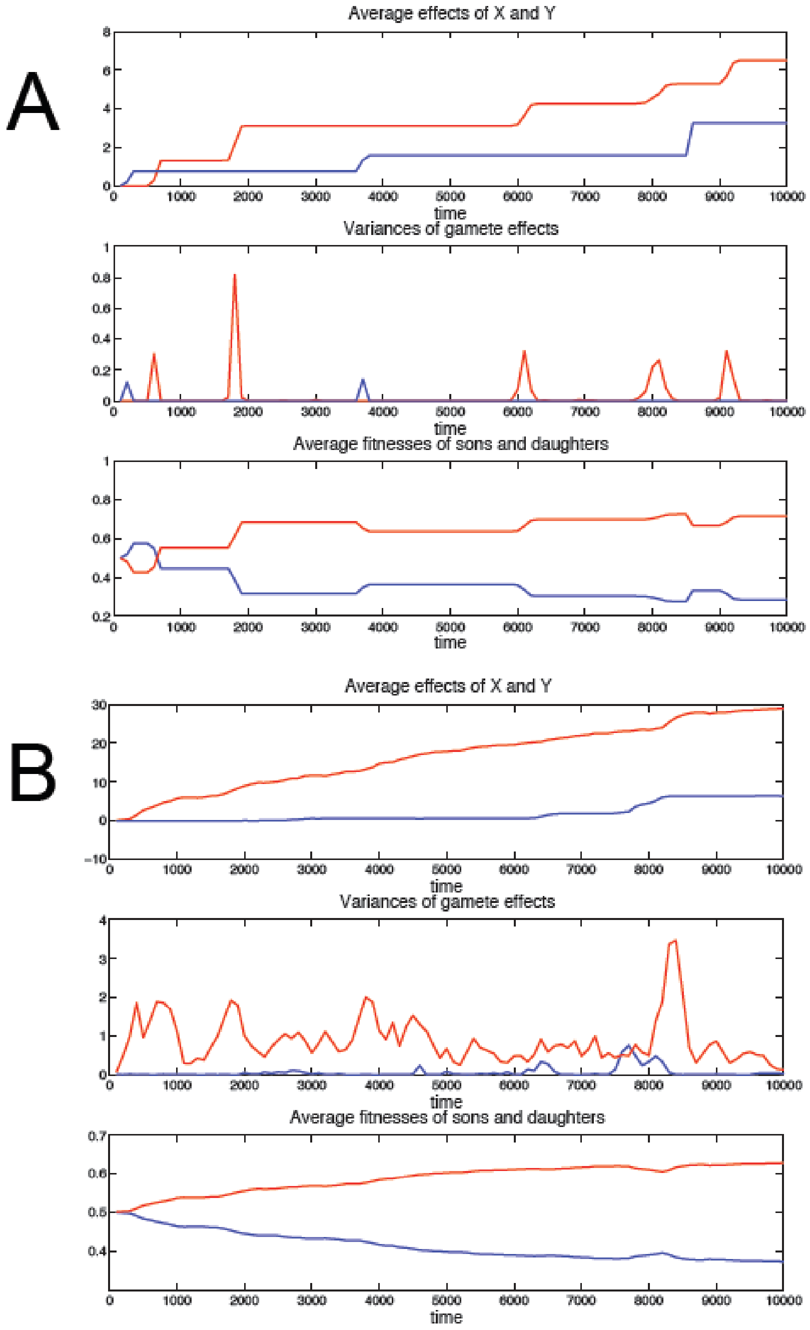

Figure S2. The effects of a reduced mutation rate in Y on the dynamics of the average values $\bar{x}$ and $\bar{y}$, variances V_x and V_y and the average fitnesses of sons and daughters W_x and W_y . (a) A run with α= 0.4 and μ = 0.00001. (b) A run with α = 0.025 and μ = 0.001. Mutation rate in Y is one tenth of that in X. Red depicts *x* and daughters and blue depicts *y* and sons.

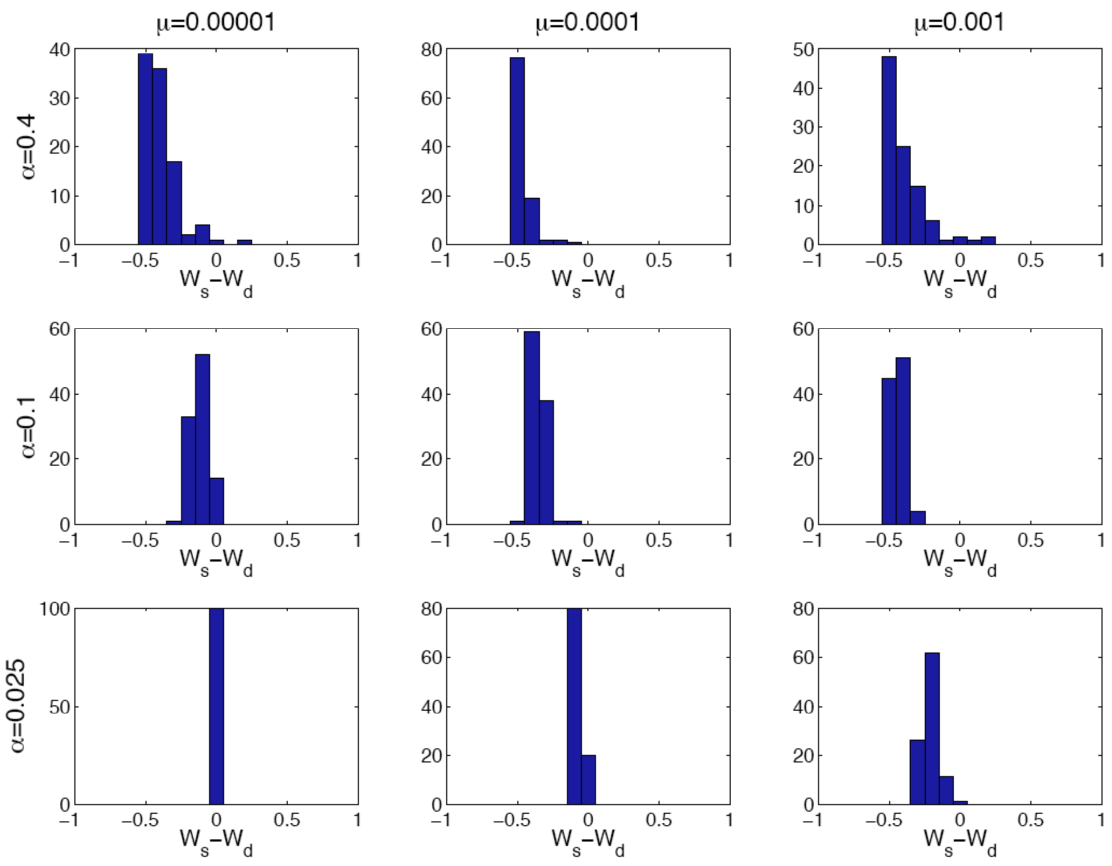

Figure S3. The effects of a reduced mutation rate of the Y on fitness skew $W_x - W_y$. 100 data points for each parameter configuration. Mutation rate on the Y is one tenth of that of the X.
